# Supplementary material for: Seminoma and Embryonal Carcinoma Footprints Identified by Analysis of Integrated Genome-Wide Epigenetic and Expression Profiles of Germ Cell Cancer Cell Lines
Source: PLoS One. 2014 Jun 2;9(6):e98330. doi: 10.1371/journal.pone.0098330 (PMC4041891; doi:10.1371/journal.pone.0098330)
Supplement: File S1 — ZIP file containing DMRforPairs output for significant regions. Please start from the html files. (ZIP) [file pone.0098330.s008.zip › figures/37276.pdf]

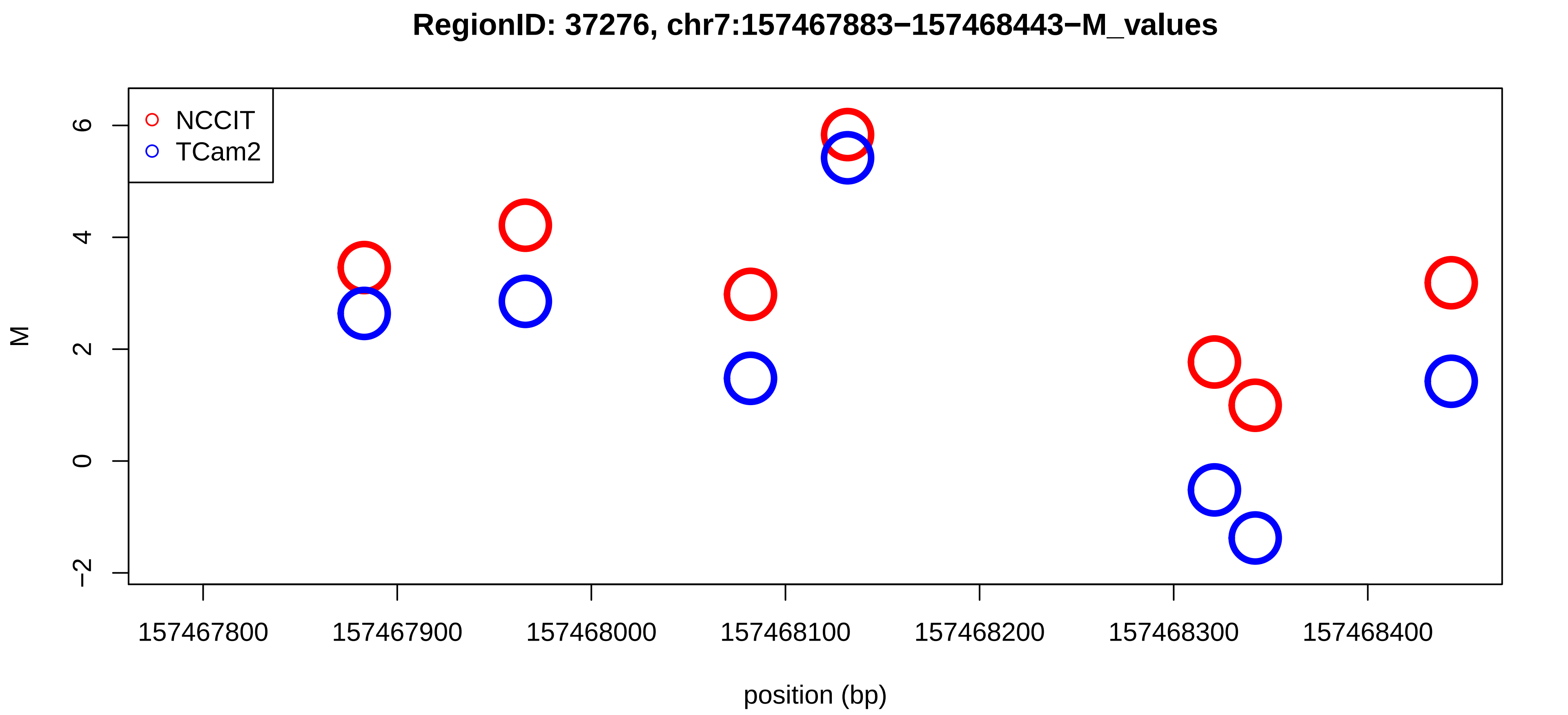

RegionID: 37276, chr7:157467883−157468443−M_values
NCCIT
TCam2
M
−2
0
2
4
6
157467800
157467900
157468000
157468100
157468200
157468300
157468400
position (bp)

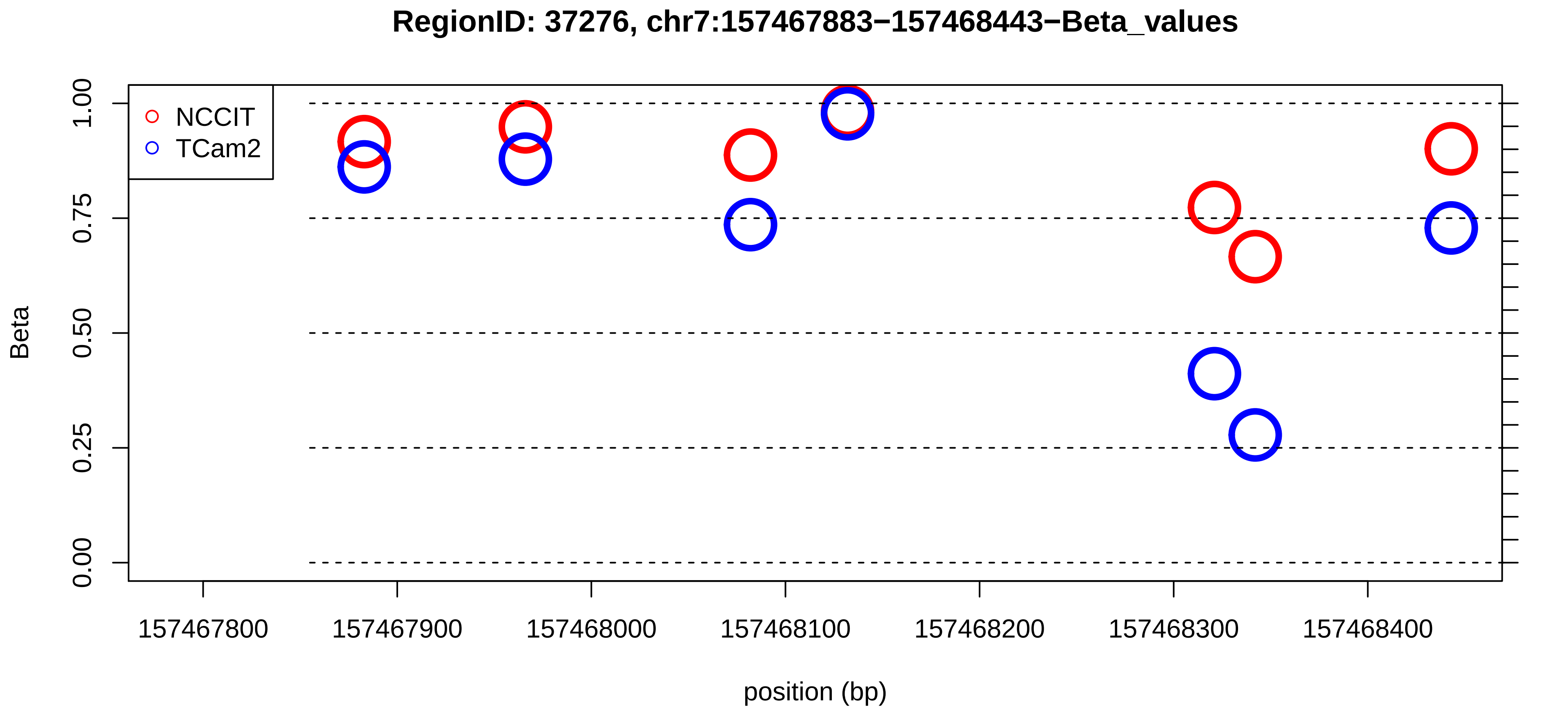

RegionID: 37276, chr7:157467883−157468443−Beta_values
NCCIT
TCam2
Beta
0.00
0.25
0.50
0.75
1.00
157467800
157467900
157468000
157468100
157468200
157468300
157468400
position (bp)
